# Comprehensive identification, characterization, and expression analysis of m^6^A methylation transferase gene family in maize (*Zea mays* L.)

**DOI:** 10.3389/fpls.2025.1673408

**Published:** 2025-10-31

**Authors:** Chunlei Li, Ming He, Xinyi Liu, Qingxue Lv, Xiaoming Yu, Xiaoxue Fan, Xia Bai, Jie Cong, Chuanbo Sun

**Affiliations:** ^1^ College of Agronomy, Jilin Agricultural Science and Technology College, Jilin, Jilin, China; ^2^ Jilin Academy of Agricultural Sciences, Changchun, Jilin, China

**Keywords:** maize, epitranscriptomic regulation, m6A modifier, evolution, expression profile, cis-acting elements

## Abstract

N6-methyladenosine (m6A) RNA represents the most prevalent internal modification found in mRNA and plays a crucial role in stress response and developmental processes across various crop species. However, the understanding of m6A modification in monocot species remains limited. In this study, we conducted a comprehensive analysis of m6A methyltransferase genes “writers” in maize, focusing on gene localization, structural characteristics, conserved domains, phylogenetic relationships, promoter analysis, and expression profiles. Our analysis identified three m6A regulatory genes within the maize genome. Through the phylogenetic relationship analysis, we classified these genes into three distinct clusters alongside the model species *Arabidopsis thaliana* and *Oryza sativa*. Promoter analysis revealed that m6A pathway genes are mainly associated with hormone-responsive elements, environmental stress-related elements, and transcription factors. The conserved domain analysis indicated that all identified maize m6A proteins possess the MT-A70 domain. Furthermore, RNA-seq and RT-qPCR analysis demonstrated that the identified Zmm6A genes exhibit tissue-specific expression patterns, as well as differential expression in response to various abiotic stresses, suggesting a potential role for m6A modification in influencing reproductive development. Notably, the expression of ZmMTA-03 genes was significantly upregulated under cold stress conditions. This study provides valuable insights into the regulatory genes associated with m6A modification and their potential epigenetic regulatory mechanisms in maize.

## Introduction

Maize (*Zea mays*) ranks first in the world in terms of total production and average yield per unit area among the three major cereals in the world (Gezahegn, 2021). China ranks as the second-largest producer of maize, following the United States of America (Zhang and Yang, 2019). It is estimated that approximately one-third of the global population depends on maize as their primary source of food in sub-Saharan Africa, Southeast Asia, and Latin America (Nuss and Tanumihardjo, 2010). Moreover, around 65% of the world’s maize is utilized as animal feed, and in developed countries, it is as high as 80%. RNA molecules play a crucial role in all living organisms. These molecules act as carriers to transfer genetic information from DNA to protein and act as regulators of various biological processes (Hu et al., 2024). RNA transcripts can undergo a range of complex chemical modifications, which facilitate the formation of structures tailored to perform specific molecular functions (Nachtergaele and He, 2018). In fact, over the last eight decades, more than 150 modifications of RNA have been found in RNA. Among them, N6-methyladenosine (m6A) is one of the most important RNA modifications and plays a vital role in different steps of mRNA function, including mRNA degradation, stability, translation, and miRNA processing in multiple species (Visvanathan and Somasundaram, 2018; Hou et al., 2022).

Methyladenosine m6A is a dynamically reversible modification consisting of three major components, including m6A methyltransferases (writers), binding protein (readers), and demethylase (eraser) (Roundtree et al., 2017; Shi et al., 2019). Writers are complex proteins, such as METTL3, which has m6A methyltransferase activity and functions as a catalytic subunit, and METTL14, which is mainly responsible for the binding of the complex target RNA (Shi et al., 2019). The m6A methyltransferase in plants is highly conserved in animals. METTL3 homologous protein MTA (adenosine methylase) and METTL14 homologous protein MTB (methyltransferase B) are core subunits that have been identified in *Arabidopsis thaliana* (Shen, 2023). m6A methyltransferase is complex, which contains the MT-A70 conserved domain (Luo et al., 2022). The formation of the m6A gene in Arabidopsis has a powerful gene knockout mutant library, which can be used as an ideal model organism for studying m6A RNA methylation. In Arabidopsis, AtMTA proteins are mainly distributed in meristems, especially in reproductive organs, apical meristems, and new roots (Yue et al., 2019). The inactivation of AtMTA proteins will hinder the development of plants, leading to the stagnation of plant embryos at the globular stage, and ultimately lead to embryo death. The m6A methylation modification reduces the relative abundance of MTA proteins in reproductive organs, buds, and lateral root meristems (Zhong et al., 2008). It has been demonstrated that N6-methyladenosine (m6A) plays a crucial role in regulating male sterility in various crops, including maize (Wan et al., 2019), and wheat (Melonek et al., 2021). In the wheat line YS3038, the expression levels of m6A in the anthers exhibit significant variation between sterile and fertile conditions. Compared with sterile conditions, the total RNA of m6A level in anthers was significantly reduced in fertile conditions. Consequently, the m6A enrichment of mRNA was significantly reduced, the number of m6A methylation sites and m6A-modified genes (Zhao et al., 2023). Male sterility is an important trait for heterosis utilization and hybrid seed vigor to improve crop yield and quality; it is one of the effective means to improve crop yield for hybrid breeding. In addition, there is evidence that m6A modifications are involved in several stress regulatory responses (Dominissini et al., 2012; Scutenaire et al., 2018; Yang et al., 2021). Moreover, recently several studies which characterized the m6A regulatory mechanism gene in tomato (Zhou et al., 2019), Camellia sinensis (Zhu et al., 2021), *Brassica rapa* (Liu et al., 2020), and strawberry (Zhou et al., 2021). Thus, in plants compared to Arabidopsis, there has been limited research done on the regulatory mechanism as well as the function of m6A modification in maize crops. In plants, and several factors that cause male sterility include: (1) natural mutation; (2) physical mutagenesis; (3) biotechnology innovation; and (4) ecological and environmental impacts. The phenomenon of maize heterosis is widely used in agricultural production. However, there are no reports on the study of m6A methylation modification in maize male sterility.

In this study, the bioinformatics analysis methods were used to systematically identify the MTA gene family of m6A methylation modification, and the systematic evolution analysis, gene structure analysis, amino acid physicochemical properties analysis, conserved domain analysis and gene-specific expression pattern analysis of MT-A70 protein family were carried out, laying a foundation for the study of the function of m6A methylation modification in maize.

## Materials and methods

### Sequence retrieval and identification of ZmMTA genes

The genome sequences and gene annotation files of *Arabidopsis thaliana* were downloaded from the plant genome database EnsemblPlants (https://plants.ensembl.org/index.html). Two m6A modifier genes from Arabidopsis, AT4G10760 (MTA) and AT4G09980 (MTB), were used as reference queries (Kersey et al., 2012). Subsequently, we employed the BLASTp method with an e-value set to 1 × 10^5 to identify orthologous proteins of MTA in the maize genome. Additionally, the identified ZmM6As were further validated by examining their conserved domains within the context of their gene family. The genome sequences and annotation information of each species were extracted using the GXF sequence extraction and FASTA Get functions in TBtools to obtain the representative CDS sequences. Protein sequences were then extracted after simplifying the names. The conserved domain of these candidate genes was predicted using the Conserved Domain Database (CDD) of NCBI (https://www.ncbi.nlm.nih.gov/Structure/bwrpsb/bwrpsb.cgi). The conserved domains were analyzed using InterPro (https://www.ebi.ac.uk/interpro/). The candidate genes with m6A-modified conserved domains MT-A70 were identified in all three MTA genes.

### Identification of protein characteristics and in-silico subcellular localization

The ExPASY proteomic online analysis tool (https://web.expasy.org/protparam/) was used to assess the physicochemical properties of ZmMTA protein. This analysis includes parameters such as the number of amino acids, molecular weight, isoelectric point, number of aliphatic amino acids, and hydrophobicity of proteins (Duvaud et al., 2021). To predict the subcellular localization of m6A proteins, the Plant-mPLoc (http://www.csbio.sjtu.edu.cn/bioinf/Cell-PLoc/) was utilized for analysis of ZmMTAs as previously described by (Chou and Shen, 2008).

### Analysis of conserved motifs, domains, and structure of ZmMTA

The MEME program (https://meme-suite.org/meme/) was utilized to identify motifs by selecting 8 motifs from a comprehensive analysis of all protein sequences (Joly-Lopez and Bureau, 2014). Subsequently, TBtools were employed to draw the conserved motif and conserved domain of ZmMTA protein (Chen et al., 2020). The structural characteristics of ZmMTA were analyzed by examining conserved motifs and the distribution of exons and introns. Genomic location data from the General Feature Format Version 3 (GFF3) of the maize genome were imported into the TBtools software to visualize the intron-exon structures. Subsequently, TBtools were employed to draw the gene structure and conserved motif of ZmMTA family protein (Chen et al., 2020).

### Phylogenetic tree analysis of m6A proteins

The protein sequences of the m6A gene from Arabidopsis, maize, and rice were retrieved. The MUSCLE was employed for the alignment of the protein sequences, respectively. Subsequently, the evolutionary tree was constructed based on the optimal model (JTT+G+I+F model) in MEGA11 through the Neighbor-Joining (NJ) method with the p-distance distribution model. A bootstrap value of 1,000 was selected. Lastly, the phylogenetic tree was visualized on the iTOL website (https://itol.embl.de/), respectively.

### Mining cis-regulatory elements of ZnMTAs promoters

The regulation of gene expression is critically dependent on cis-acting elements. To analyze these elements within the promoter sequences of the MTA gene family, the upstream 2000 bp sequences were retrieved from genomic sequence information and annotation files of maize using the GXF Sequences Extract of TBtools software, and the promoters of the target gene family members were extracted and uploaded to the PlantCare website (https://bioinformatics.psb.ugent.be/webtools/plantcare/html/) to predict the possible cis-regulatory elements in the promoter region.

### Expression analysis of ZmMTA family genes

The expression data for MTA family genes across various tissues were collected to analyze the tissue-specific expression patterns of ZmM6As. Furthermore, RNA-seq data for ZmM6A were retrieved to investigate the expression profile under different stress conditions, including cold, heat, salt, and UV light treatments, along with control conditions. The TBtools were employed to generate heat maps that visualize the expression profiles of ZmMTAs in different tissues and under various stress conditions (Xia et al., 2017).

### Chromosomal locations and collinearity analysis of ZmMTA

The chromosomal locations of the ZmMTA genes within the maize genome were determined based on their position, with coding sequences (CDS) data obtained and visualized using the GFF3 annotation file in TBtools. The genome assemblies of *Zea mays*, *Arabidopsis thaliana*, and *Oryza sativa* were employed to visualize synteny and collinearity, which were examined through a multi-synteny plot generated using the one-step MCScanX program in TBtools (Chen et al., 2020).

### Protein-protein interaction network analysis

Proteins and their interactions are crucial for biological function. To investigate this phenomenon, we utilized the STRING database (https://string-db.org/) (Szklarczyk et al., 2023), to analyze and predict the protein-protein interaction network of ZmM6As. This analysis included the protein sequencing of each gene identified in this study, specifically focusing on the organism *Zea mays*.

### Plant material and stress condition

To investigate the stress response, uniform-sized and shaped maize seeds of the B73 inbred line were selected. These seeds were disinfected with 75% alcohol for 4 minutes and subsequently rinsed several times with distilled water. The seeds were then grown in a growth chamber under a photoperiod of 16 hours of light at 26 ^○^C and 8 hours of darkness at 20 ^○^C. Salt stress was applied at the three-leaf seedling stage using 200 mM NaCl, following the methodology described by (Cai et al., 2017). Following this, heat stress was subjected to the seedlings in the growth chamber at 43^○^C, as outlined (Shi et al., 2017). Additionally, the maize seedlings were subjected to cold stress at 5^○^C (day/night) in accordance with (Hu et al., 2025). Leaf samples were harvested after 24 hours of treatment and instantly frozen for all treatments, then stored at -80^○^C for RNA extraction.

### RT-qPCR

Total RNA was extracted using RNAiso Plus (Takara, Japan) in accordance with the manufacturer’s protocols. Following the extraction, DNase treatment was conducted to eliminate any contaminating DNA. Reverse transcription was then performed using the Union Script first-strand cDNA synthesis mix. Then, the resulting cDNA served as a template for RT-qPCR, which was performed using the SYBR Green Fast Mix Real-Time PCR System. The RT-qPCR reactions were carried out as described by (Guan et al., 2025), with three biological replicates for each sample. The gene expression level was normalized to the expression of the β-action and quantified using the 2-^^^CT^ method. Specific primer information is available in the **Supplementary Table S1**.

## Results

### Identification and staining localization of ZmMTA protein family genes

To identify the homologs of Atm6As and Osm6As in maize, a BLAST and CD-search analysis was conducted. Following the alignment of sequences and the conformation of conserved domains, three genes were identified and aligned with the maize genome, subsequently renamed as ZmMTA-01, ZmMTA-02, and ZmMTA-03 as detailed in **Table 1**. The physicochemical properties and subcellular localization analysis of the ZmMTA family proteins were examined utilizing the ExPASY proteomic online tool. The ZmMTAs genes in maize encode proteins are characterized by diverse amino acid compositions, physicochemical properties, distributions, number of amino acids, molecular weights, isoelectric points, fat coefficients, and average hydrophobicity, as summarized in **Table 2**. Overall, ZmMTA-01 has the highest amino acid content, while ZmMTA-02 has the lowest. The fat coefficient of ZmMTA proteins ranges from 50 to 80, indicating higher stability. In maize, the hydrophobicity analysis of m6A-modified proteins indicates negative values, suggesting that the ZmMTA family comprises exclusively hydrophilic proteins. Subcellular localization reveals that ZmMTA-01 and ZmMTA-03 are localized in the nucleus, whereas ZmMTA-02 is associated with the chloroplast. This localization pattern implies that the primary function of the ZmMTA protein is within the cell nucleus. Additionally, given that chloroplasts possess their own independent DNA capable of replication and transcription. The presence of ZmMTA protein in chloroplasts may suggest a regulatory role that influences both the development and photosynthetic functions of these organelles (Maple and Møller, 2007).

**Table 1:** Description of m6A-related genes in maize.

Gene ID	Name	Character	OFR name	Accession in Uniport
Zm00001d027671	MTA-01	Methyltransferase -like protein 1	ZEAMMB7_ Zm00001d027671	A0A1D6JNR0
Zm00001d017566	MTA-02	N6-adenosine -methyltransferase MT-A70-like protein	ZEAMMB73_Zm00001d017566	B6SVX1
Zm00001d028128	MTA-03	DNA glycosylase superfamily protein	ZEAMMB73_Zm00001d028128	A0A1D6JS95

**Table 2:** Summary of physicochemical properties and subcellular localization of m6A-related genes in maize.

Transcript id	Gene name	Gene location	A.A Length	PI	Molecular weight (kDa)	Subcellular localization	Gravy	Aliphatic index
Zm00001d027671	MTA-01	Chr1, 10616170.10621462	835	6.76	94721.56	Nucleus	-1.103	54.28
Zm00001d017566	MTA-02	Chr5, 198723181.198729267	418	8.93	48168.12	Chloroplast	-0.526	79.31
Zm00001d028128	MTA-03	Chr1, 23407593.23418703	704	6.93	77861.34	Nucleus	-0.392	82.77

### Conserved motifs, domains, and gene structure analysis

**Figure 1:**
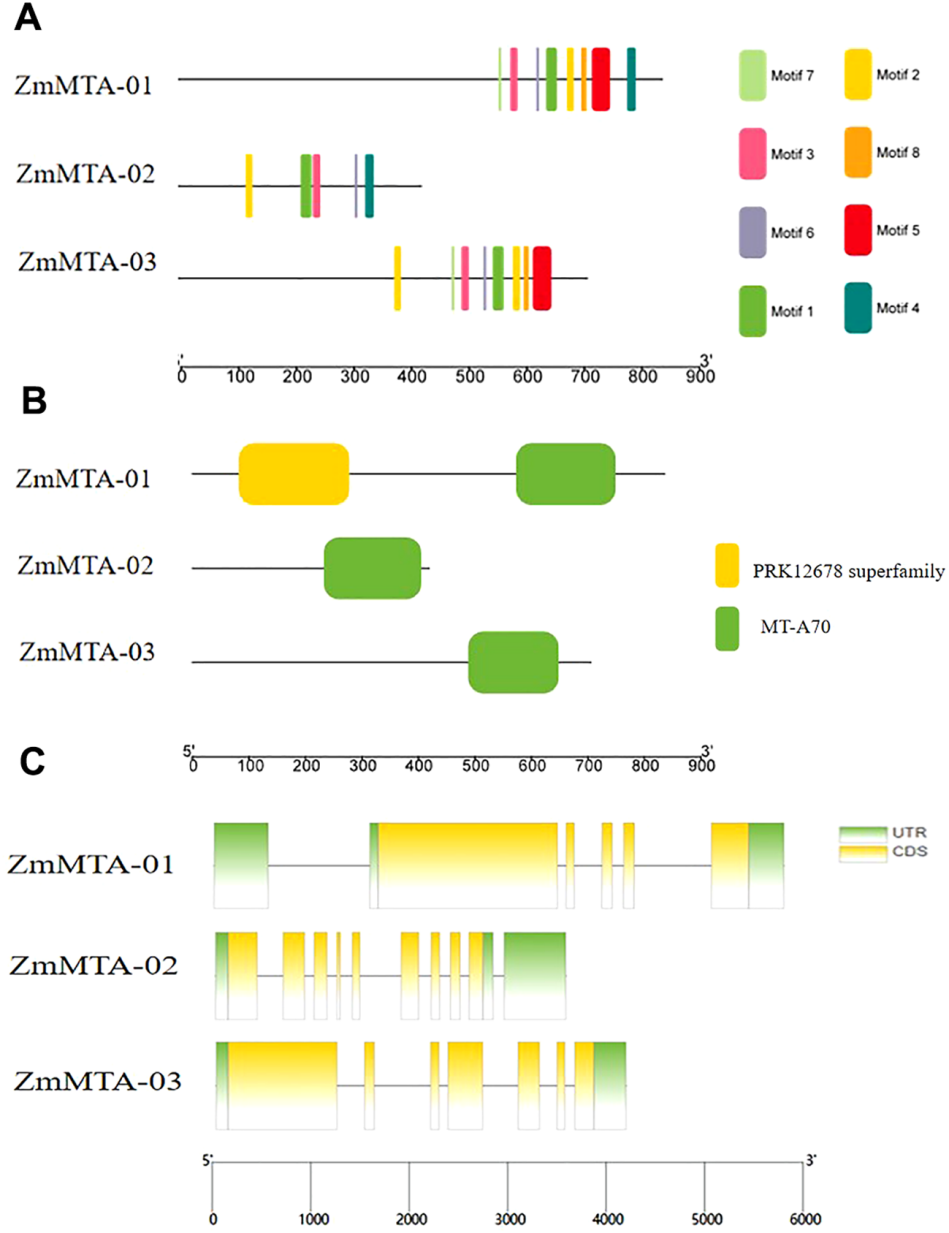
**(A)** The composition of ZmM6A protein motifs is illustrated using a color-coded scheme for motifs 1 through 8. **(B)** A visualization of the conserved domains within ZmM6A proteins. **(C)** The arrangement of exons and introns in ZmMTA genes is depicted, with yellow boxes representing exons and black lines denoting introns. The relative positions of these features are proportionally represented on a kilobase scale at the bottom of the figure.

To identify the motifs present within each identified m6A protein. The motif analysis revealed that ZmMTA-01 and ZmMTA-03 contained all eight motifs, whereas ZmMTA-02 exhibited only five motifs. These results indicate significant variability in the presence of short motifs among the ZmMTA proteins (**Figure 1A**). The m6A methyltransferases in Arabidopsis are characterized by the presence of a conserved MT-A70 domain (Luo et al., 2022). Our analysis of the conserved domains in maize MTA gene family proteins demonstrated similarities to the AtMTA and OsMTA gene families, both of which also feature the highly conserved MT-A70 domain (**Figure 1B**). Furthermore, ZmMTA-01 contains a PRK12678 superfamily domain; the function of which remains to be discovered. These findings suggest that the maize m6A ZmMTAs possess fewer domains beyond the MT-A70, indicating potential functional redundancy among these proteins. Additionally, this study investigated the gene structure of the ZmMTA gene family, as visualized in **Figure 1C**. It was found that the gene family consists of 6 to 12 exons, with ZmMTA-01, ZmMTA-02, and ZmMTA-03 containing untranslated regions (UTRs) at both 5‘ and 3‘ ends.

### Phylogenetic analysis of ZmMTA protein family proteins

**Figure 2:**
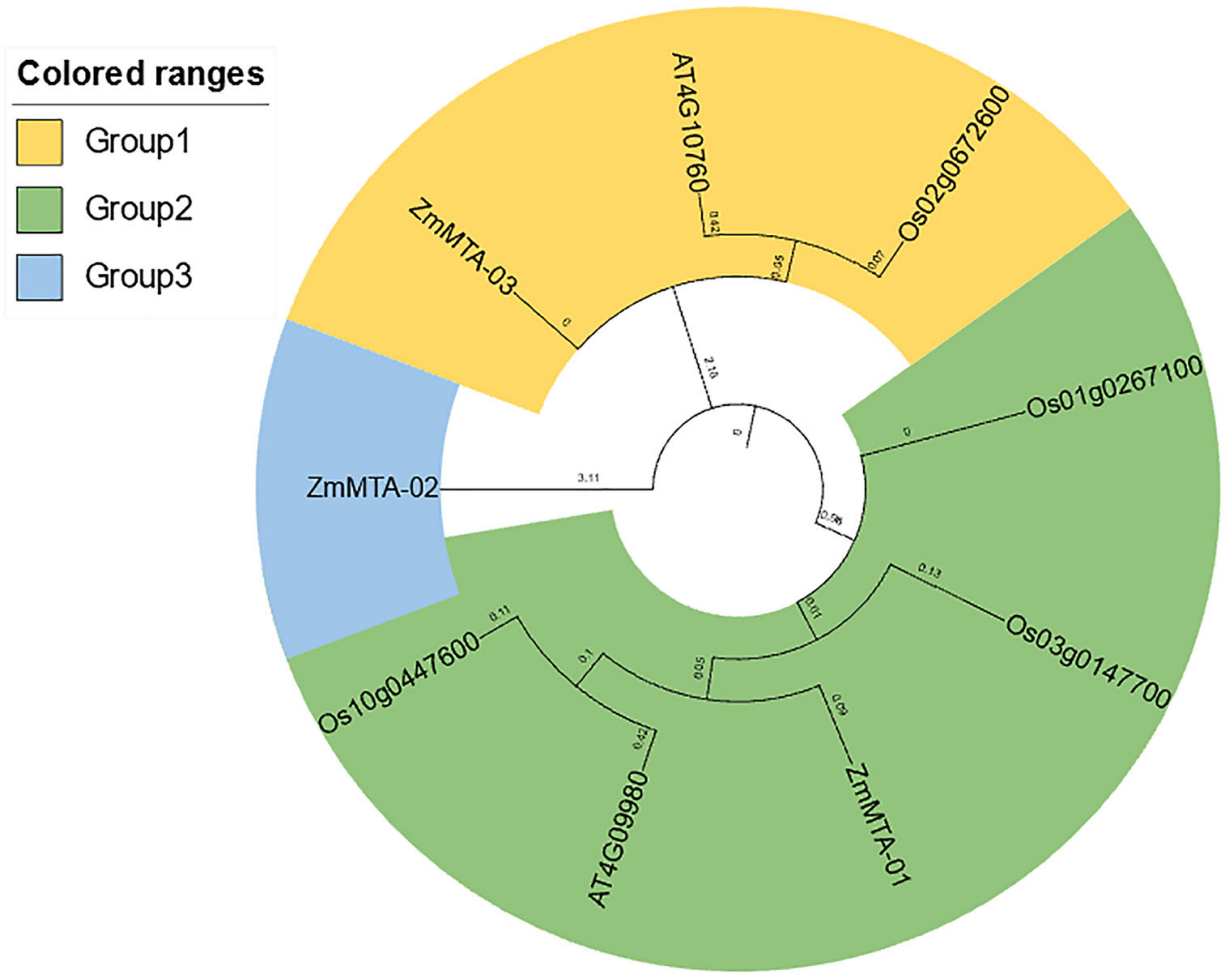
Phylogenetic tree depicting m6A-related genes in maize, with distinct evolutionary clades represented by various colors.

A phylogenetic tree was constructed to analyze the m6A proteins from maize, rice, and Arabidopsis. This phylogenetic analysis involved three distinct clusters as groups, which included two proteins from Arabidopsis, four proteins from rice, and three proteins from maize. Group 1 includes one Arabidopsis MTA (AT4G10760) protein, one rice MTA (Os02g0672600), and one maize MTA (ZmMTA-03). Group 2 consists of one Arabidopsis MTA (AT4G09980), three rice MTAs (Os01g0267100), (Os03g0147700), (Os10g0447600), and one maize MTA (ZmMTA-01). Group 3 consists of only one maize MTA (ZmMTA-02) (**Figure 2**). Notably, except for (ZmMTA-02), the other two maize MTA proteins did not exhibit significant sequence divergence from AtMTA protein of Arabidopsis and OsMTA proteins of rice.

### Prediction of cis-acting elements in promoters of ZmMTA family genes

**Figure 3:**
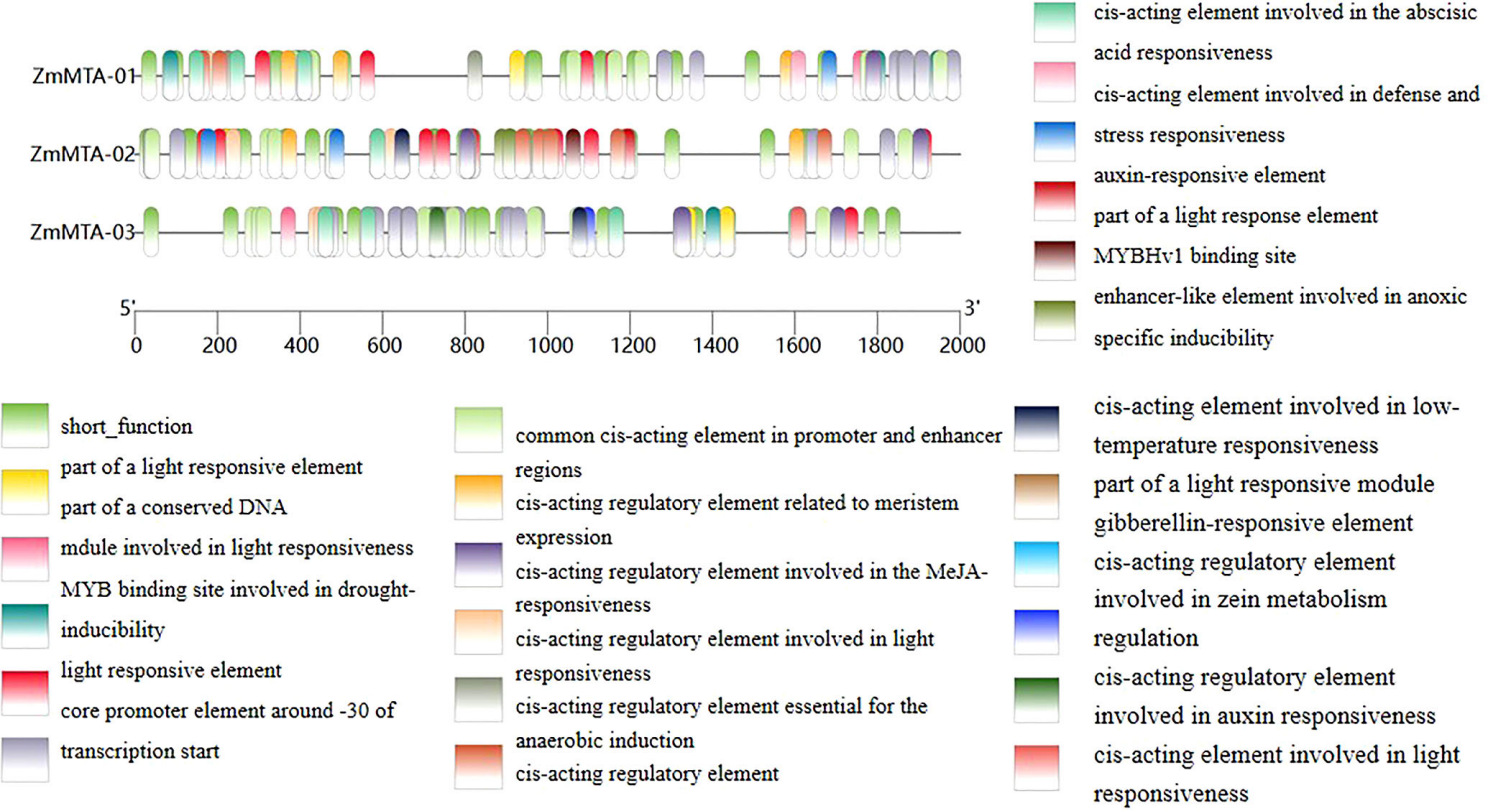
Analysis of cis-acting elements in the ZmMTA genes was conducted within the 2000 bp upstream region of its promoter sequences. The classification of cis-regulatory elements was based on their functional significance, utilizing the PlantCARE database.

The promoters of the ZmMTA gene family were analyzed, and the results are shown in **Figure 3**. A total of 24 cis-regulatory elements were identified in the promoter region of the maize MTA gene. It is worth noting that these elements include cis*-*acting elements such as jasmonic acid (MeJA), auxin (IAA), abscisic acid (ABA), gibberellin (GA), and zeatin photoperiod response elements. Among them, the three MTA genes all contain light response-related elements, indicating that the ZmMTA gene may change its expression level due to light signals, thereby participating in the regulation of maize reproductive development and the light response process.

### Analysis of tissue-specific expression of ZmMTA genes

**Figure 4:**
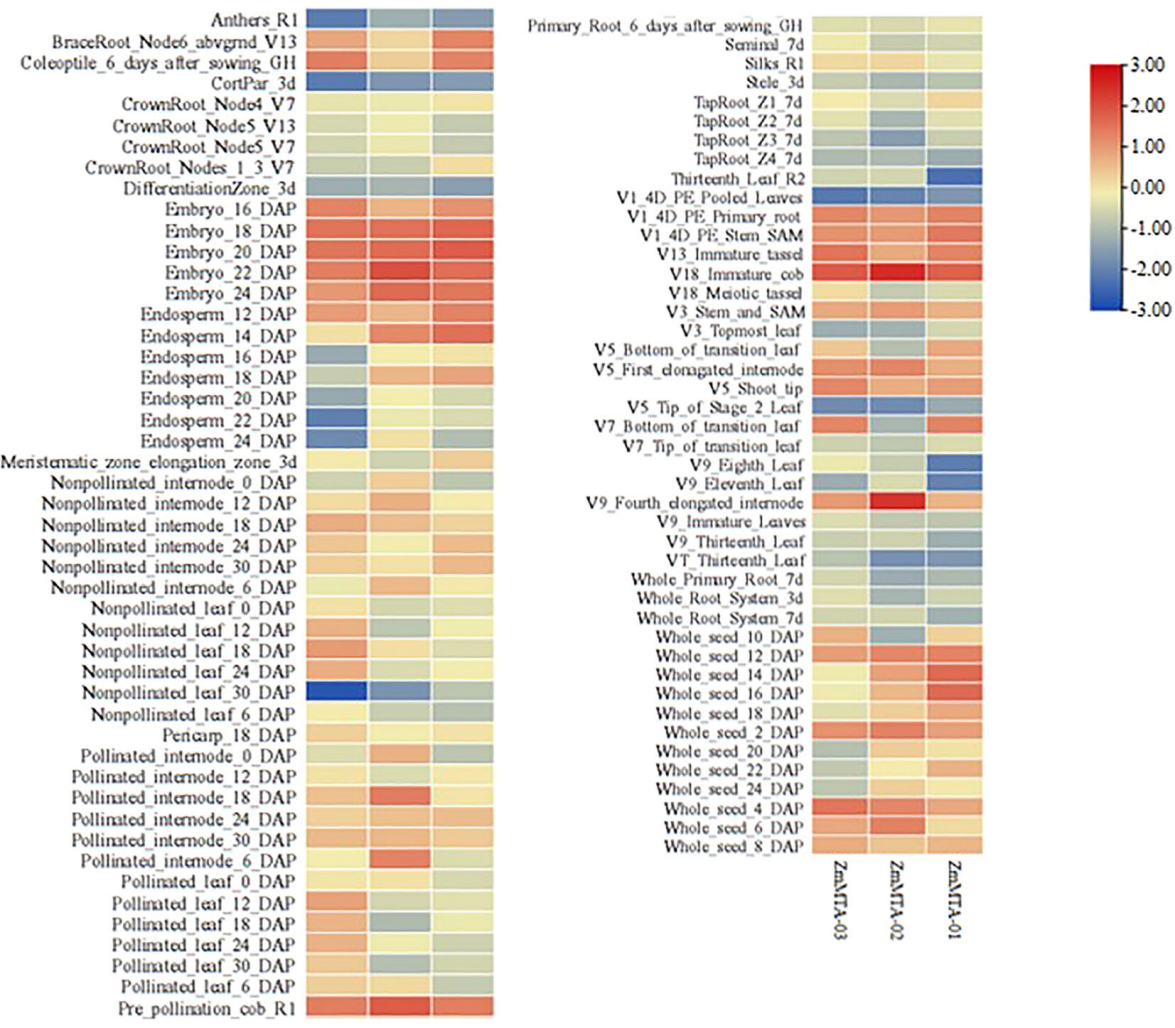
The analysis of tissue-specific expression of maize MTA genes indicated that higher expression levels are indicated by red boxes, while lower expression levels are denoted by blue boxes.

The expression patterns of ZmMTA family proteins were analyzed by utilizing maize transcriptome sequencing data obtained from the maize database (https://qteller.maizegdb.org/genes_by_name_B73v5.php). Initially, the expression characteristics of ZmMTA genes across various tissues during both vegetative and reproductive stages were examined, and the results are shown in **Figure 4**. All ZmMTA genes were found to be expressed in at least one of the tested tissues. Notably, three ZmMTA genes exhibited expression during the early stages of embryo development; however, expression levels decreased 16 days post-pollination, particularly in the endosperm, where expression was notably lower. Furthermore, the ZmMTA genes showed high expression levels in both female and male ears, while expression in mature pollen was minimal. Additionally, expression levels were low during certain developmental stages of roots and leaves. These observations suggest that ZmMTA may play a significant role in the early development of maize embryos as well as in the growth and development of immature reproductive organs.

**Figure 5:**
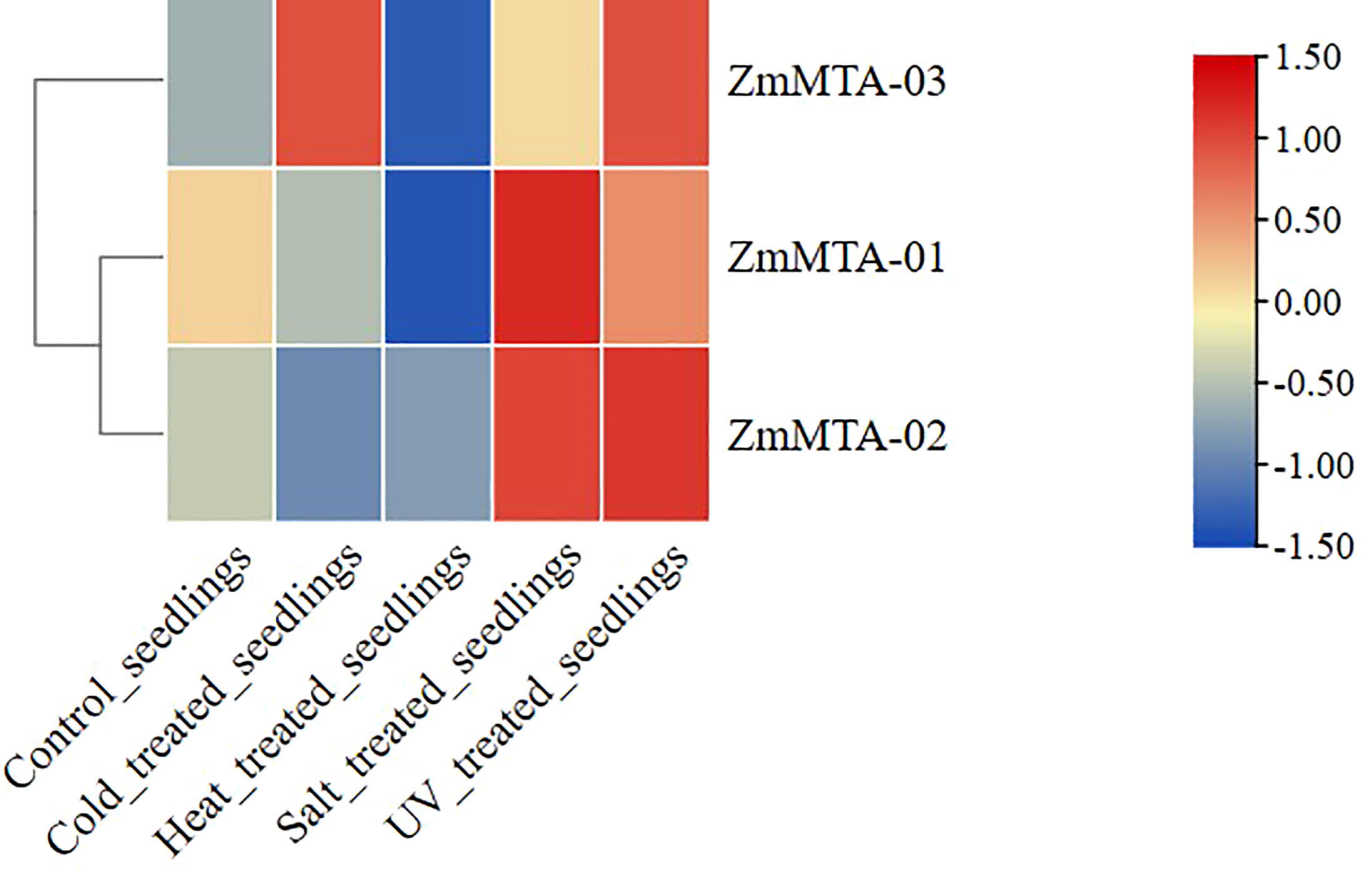
Expression patterns of maize MTA genes under abiotic stress conditions indicated that the increased expression levels are denoted by red boxes, while decreased expression is denoted by blue boxes.

The expression characteristics of ZmMTA in response to environmental stressors were also investigated and illustrated (**Figure 5**). The expressions of ZmMTA-01 and ZmMTA-02 were comparable, exhibiting high expression levels under salt stress and ultraviolet irradiation, while the expression under cold and heat stress demonstrated lower levels. Conversely, the ZmMTA-03 gene showed a high expression level in response to ultraviolet irradiation and cold stress conditions, and a low expression level under heat stress. These findings indicate that the ZmMTA genes are sensitive to various environmental conditions and may be involved in the environmental response mechanism of maize, with functional differentiation observed among the three ZmMTA genes.

### Chromosomal location and multi-synteny analysis of the ZmMTA gene

**Figure 6:**
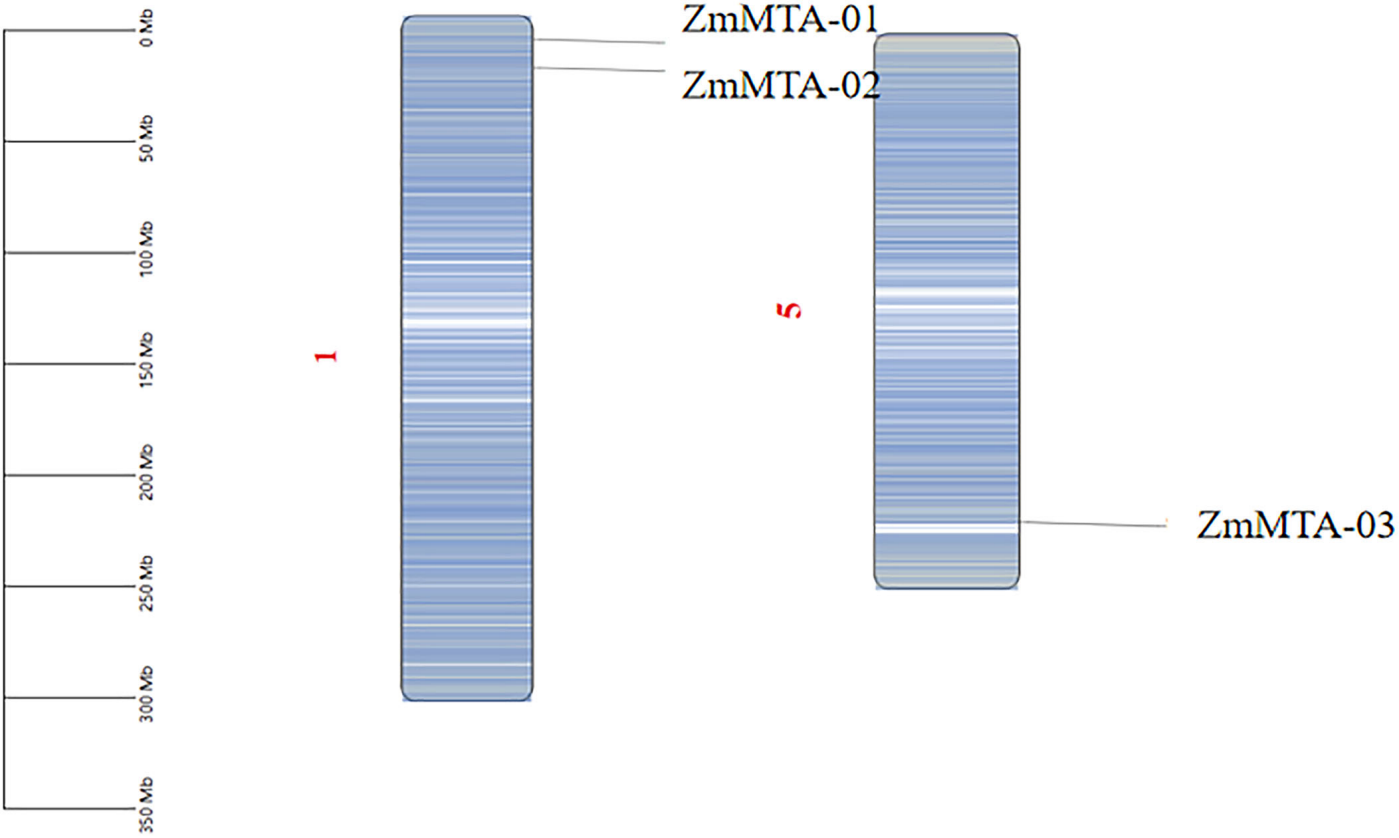
Genomic localization and distribution patterns of the ZmMTA gene within the genome are illustrated, with a gradient from blue to white indicating density ranging from low to high, while the scale on the left represents the length of chromosomes (Mb).

To elucidate the organization of the genome and the distribution of m6A-related genes in maize, a chromosomal map was constructed using TBtools. A total of three m6A-related genes were identified on the maize genome, in which ZmMTA-01 and ZmMTA-02 are located on chromosome 1, while ZmMTA-03 is on chromosome 5 (**Figure 6**). Our results revealed that the two ZmMTA genes are localized at the end of chromosome 1, and there is only one ZmMTA gene on chromosome 5. The gene duplicate identified in our study is comparable to that of m6A genes in Arabidopsis (2) and rice (4). This similarity indicates that the number of MTA genes in maize, Arabidopsis, and rice has been evolutionarily conserved.

**Figure 7:**
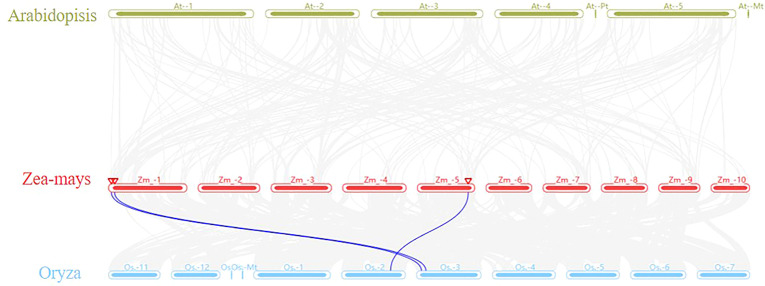
Genome-scale multi-synteny plot analysis among the genomes of Arabidopsis, rice, and maize, focusing on syntenic MTA (m6A) orthologous genes, the grey line represented the collinear blocks shared among these three species.

Furthermore, an in-depth comparative analysis of m6A was conducted using a synteny plot, through TBtoools software, focusing on Arabidopsis, rice, and maize. The findings indicated a clear collinearity between the MTA genes of maize and rice (**Figure 7**). A total of three pairs of collinear genes were identified: MTA-01 (Zm00001eb003840) was collinear with Os10g0447600 and Os03g0147700; MTA-02 (Zm00001eb007930) was collinear with Os03g0198900-01; and MTA-03 (Zm00001eb250310) was collinear with Os02g0672600. However, no collinearity was found between the maize MTA gene and the Arabidopsis MTA gene, which indicates that the MTA gene had evolved independently before the differentiation of monocots and dicots.

### Prediction of protein-protein interaction network analysis

**Figure 8:**
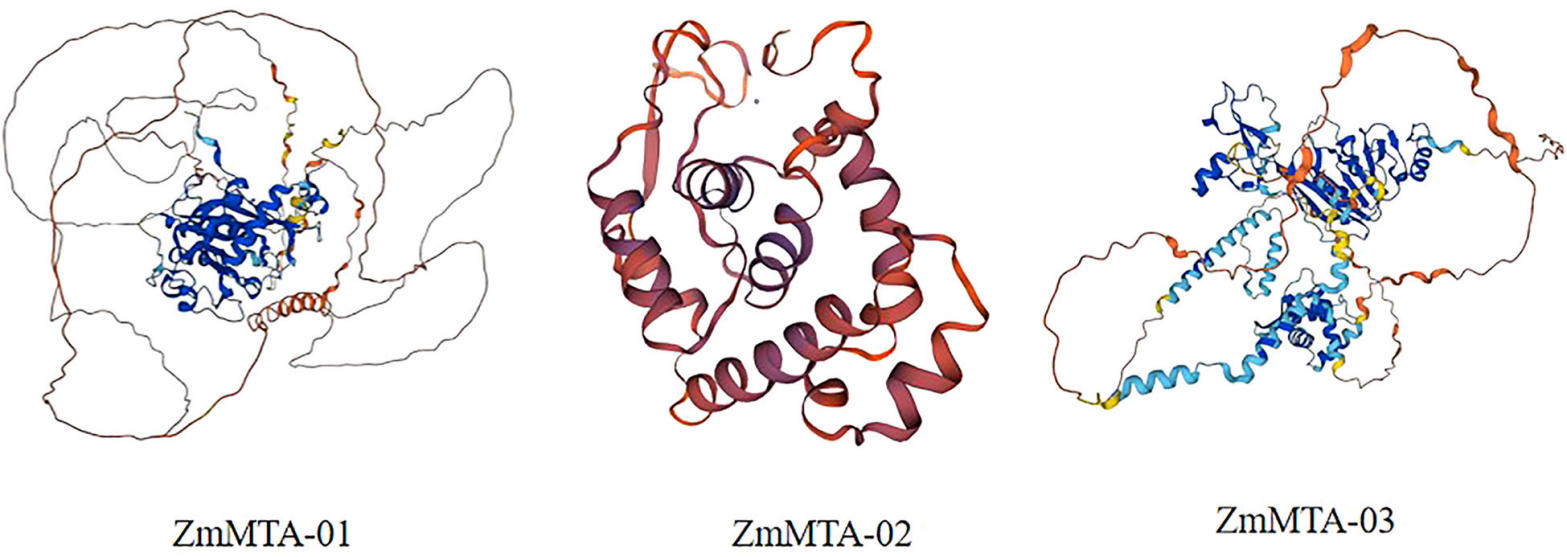
The tertiary three-dimensional structure of three ZmMTA proteins was identified within the maize genome.

**Figure 9:**
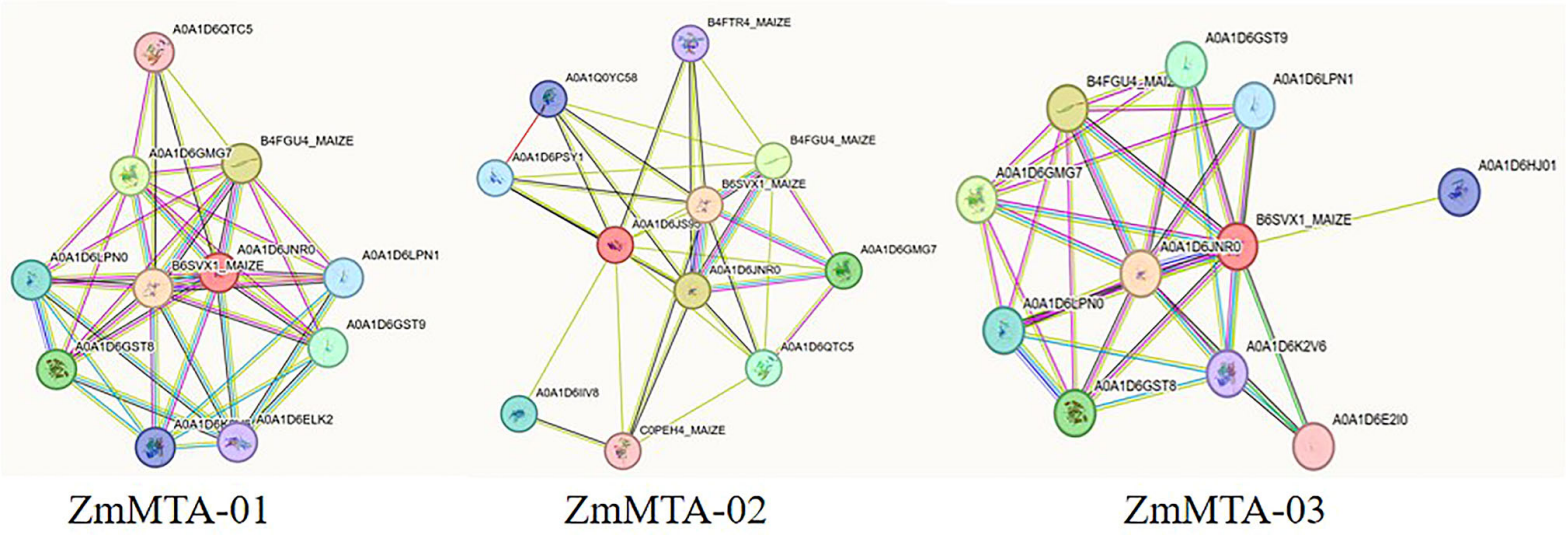
The analysis of protein-protein interaction network for ZmMTA proteins. The interaction network is categorized based on the types of protein interactions.

To enhance the understanding of the ZmMTAs protein family and their structural characteristics, a three-dimensional (3D) model was generated based on three ZmMTA genes (**Figure 8**). The 3D structures of ZmMTA-01 and ZmMTA-03 exhibit notable similarities, particularly in their core regions. Furthermore, we investigated the gene interaction network utilizing the STRING database. The results revealed that ZmMTA-01 interacts with proteins such as B6SVX1_MAIZE, B4FGU4_MAIZE, and A0A1D6GMG7. Whereas ZmMTA-02 show interactions with B6SVX1_MAIZE, A0A1D6JNR0, and B4FGU4_MAIZE. ZmMTA-03 demonstrated significant associations with proteins such as A0A1D6JNR0, B4FGU4_MAIZE, and A0A1D6GMG7 (**Figure 9**). Notably, among these interactions, the relationship between ZmMTA-03 and A0A1D6JNR0 is particularly significant. The A0A1D6GMG7 plays a crucial role in embryonic development. These findings suggest that ZmMTAs are involved in reproductive developmental processes.

### Expression patterns of m6A under abiotic stress in maize

**Figure 10:**
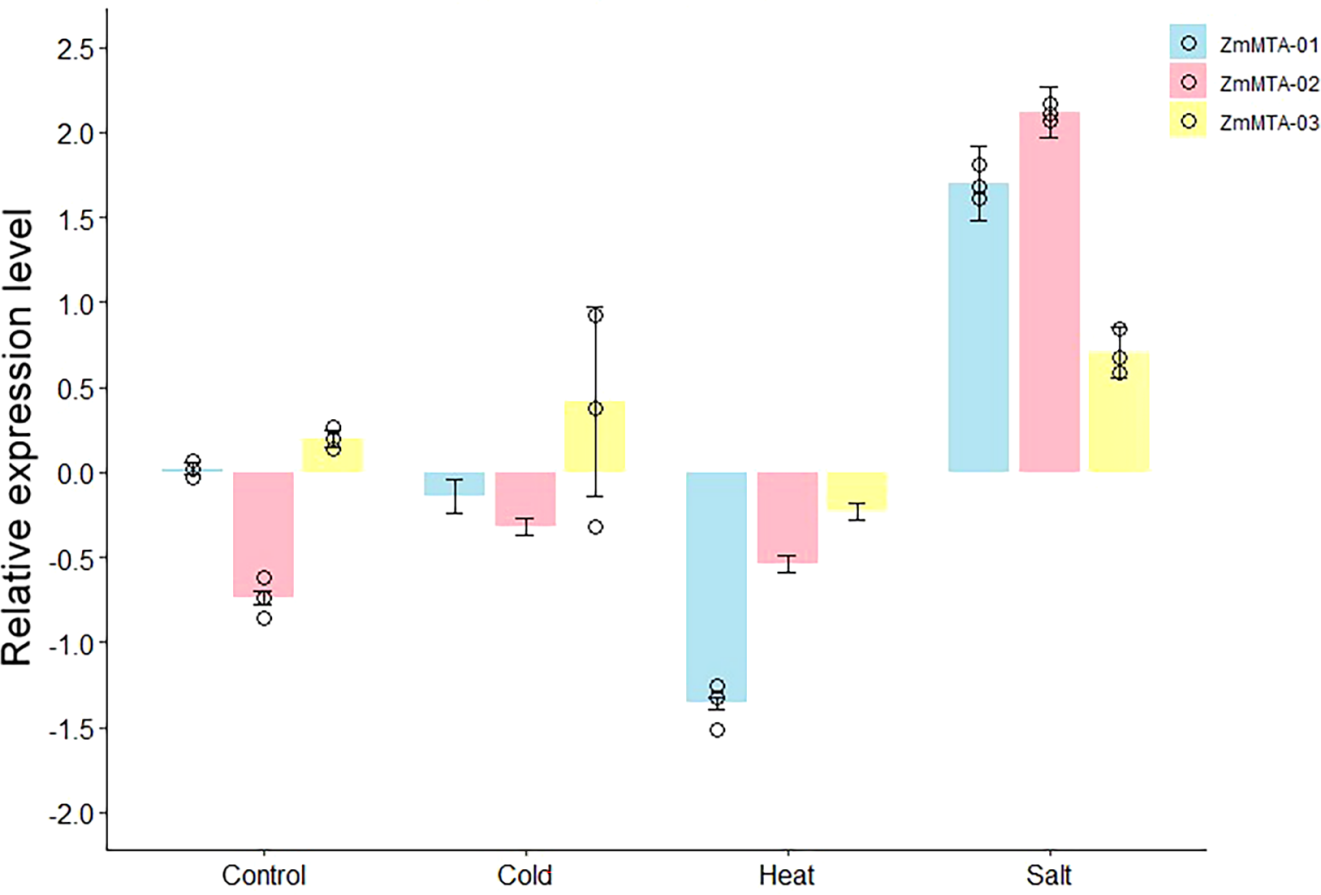
The relative expression levels of three ZmMTAs after various stress treatments were determined by RT-qPCR. The β-action gene served as an internal control.

It has been well documented that the m6A-modifier genes are involved in plant response to abiotic and biotic stress, particularly in response to cold, heat, and salt (Li et al., 2025). To investigate the expression patterns of m6A-related genes in maize under abiotic stress, we selected three genes, namely ZmMTA-01, ZmMTA-02, and ZmMTA-03, and examined their expression patterns by RT-qPCR after exposure to cold, heat, and NaCl stress. The RT-qPCR results revealed that the expression of most m6A-related genes shows a tendency consistent with the transcript abundance observed in RNA-seq. Under cold stress, ZmMTA-01 expression was significantly downregulated, while ZmMTA-03 was highly upregulated (**Figure 10**). Consequently, ZmMTA-02 shows moderate downregulation in response to cold stress. Heat stress consistently reveals the downregulation of all selected genes, showing the most pronounced expression. The expression patterns of ZmMTA-01 and ZmMTA-02 under salt stress (NaCl) were highly upregulated, thus ZmMTA-02 showing slightly higher expression than ZmMTA-01. Furthermore, the expression of ZmMTA-03 under salt stress was also positive but was less pronounced compared to other genes. These results underscore that RT-qPCR confirmed the three genes are differentially regulated in response to abiotic stress.

## Discussion

Due to environmental changes, plants are exposed to various stresses during their growth and development (Khanzada et al., 2020; Wassan et al., 2021; Solangi et al., 2023). The significant ecological consequence of such stress is male sterility in higher plants, which has profound implications for reproduction and crop productivity. This phenomenon is regulated by the coordinated expression of multiple genes. Consequently, mutations that alter the structure or function of any of these genes can lead to male sterility. Notably, over 200 nuclear male sterile mutants have been identified in maize (Luo et al., 2022). m6A methylation represents the most prevalent type of eukaryotic mRNA modification in plants (Zheng et al., 2020). Both embryonic and post-embryonic development significantly influence plant growth and stress responses, including susceptibility to various stressors (Arribas-Hernández and Brodersen, 2020). The mutant deficient in MTA or its core components of the methyltransferase complex, such as MTB, FIP37, and VIR, exhibit embryonic lethality. This observation underscore the critical functions of m6A methylation do not influence each other’s expression at the transcriptional level (Vespa et al., 2004; Zhong et al., 2008; Růžička et al., 2017; Zhang et al., 2022; Shen, 2023). The identification and functional exploration of m6A-related proteins can provide genetic information, including their theoretical foundation for enhancing crop quality and productivity (He et al., 2018; Solangi et al., 2020). Furthermore, the advancement in NGS, including genome assembly and their sequences, enabled the acquisition of vibrant genomic resources for many crop species, flagging the way for the identification of numerous gene families (Rasheed et al., 2018; Sun et al., 2022).

This study characterizes gene family related to m6A (ZmMTAs), examining their conserved domains, structures, chromosomal distribution, cis-regulatory elements, expression patterns across different tissues, responses to environmental stressors, and interactions within protein networks. A gene family is defined as a group containing more than two gene copies, indicative of gene duplication (Innan and Kondrashov, 2010). In this study, three MTA genes were identified within the maize genome through comparative genome-wide identification and expression analyses. Comparative phylogenetic analysis classified these genes into three distinct groups encompassing MTA members from Arabidopsis, rice, and maize. Notably, group three contained only one maize MTA gene, while the other two groups included MTA genes from Arabidopsis, rice, and maize. This distribution suggests that the differentiation of MTA members predates the speciation event of Arabidopsis, rice, and maize. The maize MTA genes are organized within the same phylogenetic their Arabidopsis and rice counterparts, implying that the functional roles of m6A methylase activities in maize MTA genes share similarities with those observed in our study. Moreover, the conserved domain analysis showed that ZmMTA-01 possesses both a canonical MT-A70 domain and a PRK12678 superfamily domain, indicating the possible functional diversification. Although the role of PRK12678 remains hypothetical, its co-occurrence with MT-A70 in maize may imply the neofunctionalization of these proteins within this lineage. In contrast, the protein sequence of ZmMTA-02 demonstrates significant divergence from the other two, with ZmMTA-02 uniquely localized within chloroplasts. It is hypothesized that ZmMTA-02 may have a specific role in chloroplasts (Manavski et al., 2021; Vicente et al., 2023). Consequently, it remains unclear whether ZmMTA-02 possesses m6A methyltransferase activity or other functions, highlighting the need for further research in this area.

In this study, the collinear analysis of the ZmMTA gene family in rice reveals a distinct collinear relationship between Arabidopsis and maize. Notably, the ZmMTA-01 and ZmMTA-03 genes, which are located on maize genome chromosome 1 of maize and chromosome 3, respectively, with their positional arrangement being adjacent. Consequently, these results suggest that the m6A gene in Arabidopsis may be orthologous to ZmMTA-01 and ZmMTA-03. These findings are significant for understanding the evolutionary relationships among dicot and monocot species and provide valuable insights for functional predictions. This study elucidates the potential functions and likely epigenetic regulatory mechanisms of the ZmMTAs (m6A) genes, offering critical insight into evolutionary trajectories of these genes in maize and other crop species.

Cis-regulatory elements have been extensively shown to regulate gene expression, including tissue specificity, which is crucial for appropriate development and response to stress (Biłas et al., 2016; Marand et al., 2023). It has been demonstrated that cis-regulatory elements are responsible for improving crop quality and productivity (Chen et al., 2018; Xu et al., 2024). In this study, we analyzed the cis*-*acting elements of ZmMTAs to enhance our understanding of their regulatory mechanisms and the expression of genes associated with plant development and stress tolerance. This encompasses both abiotic and biotic stress, which may serve as valuable resources for future rapid breeding strategies to improve crop productivity. The presence of cis-regulatory elements in three ZmMTA genes revealed several specific cis-acting elements unique to each gene, including MYB binding sites involved in drought stress, light-responsive elements, and those associated with phytohormones such as ABA, auxin, and MeJA. Consequently, it can be inferred that these genes are likely involved in physiological processes related to environmental stress resistance. Furthermore, in this study, the RT-qPCR analysis of three identified genes showed a strong correlation with the differential expression patterns observed in RNA-seq. The significant downregulation of all three genes under heat stress suggests a common regulatory mechanism in response to heat stress. Conversely, the diverse responses to cold and salt stress, with both upregulation and downregulation observed across the genes, highlight their potential role in specific, distinct stress-response pathways. For instance, the strong upregulation of ZmMTA-03 during cold stress underscores its potential involvement in cold acclimation. Subsequently, the high expression of ZmMTA-02 in response to salt stress suggests its unique function in mitigating osmotic stress or ion toxicity. These findings provide a compelling basis for future functional studies to elucidate their precise role in stress tolerance in maize.

## Conclusion

In this study, a genome-wide investigation and comprehensive bioinformatics analysis were employed to examine the maize m6A proteins. This study includes protein function prediction, elucidation of evolutionary relationships, analysis of conserved domains, and assessment of the subcellular localization characteristics of the ZmMTA gene. In conjunction with the findings, three m6A-related genes, ZmMTA-01, ZmMTA-02, and ZmMTA-03, were identified. All three ZmMTA genes exhibit a conserved domain, MT-A70. Subsequently, these identified ZmMTA genes underwent collinearity analysis in relation to two crop species, *Oryza sativa* and *Arabidopsis thaliana*. These results suggest that the ZmMTA gene evolved independently before the divergence of monocot and dicot species. Furthermore, analysis of tissue expression characteristics, protein structure prediction, and promoter cis-acting element prediction was conducted for the three ZmMTA genes. Our findings indicate that the ZmMTA genes are significantly expressed during the growth and development of both female and male ears. It is preliminarily speculated that ZmMTA plays a vital role in regulating the growth and development of maize reproductive organs. The results of this study provide a theoretical foundation for maize breeding, which is critical for understanding the functional implications of m6A methylation modification.

## Data Availability

The original contributions presented in the study are included in the article/**Supplementary Material**. Further inquiries can be directed to the corresponding authors.
