# Supplementary material for: Comprehensive identification, characterization, and expression analysis of m6A methylation transferase gene family in maize (Zea mays L.)
Source: Front Plant Sci. 2025 Oct 31;16:1673408. doi: 10.3389/fpls.2025.1673408 (PMC12617223; doi:10.3389/fpls.2025.1673408)
Supplement: Supplementary file 1 [file Table1.docx]

**Supplementary Table S1.** Description of the m6A-related gene primers used in RT-qPCR

| **Gene name** | **Forward primer** | **Reverse primer** | **Product length BP** |
| --- | --- | --- | --- |
| ZmMTA-01 | TGGAGCTCGAGGTGTTGATG | GGACCACCTAACCCAGTGTC | 153 |
| ZmMTA-02 | TACCATGGTTGCCGTCTGTC | TTGAGGAGTTTGCTCCGGTC | 282 |
| ZmMTA-03 | GCAAGCGTAATCAGAAGCCG | GGGGGTTCCGGAAGCAAATA | 267 |
